# The dilemma between fertility and work: How did the Universal Two-Child policy affect Chinese women’s labor income?

**DOI:** 10.1371/journal.pone.0308709

**Published:** 2024-08-08

**Authors:** Yi Chen, Zu Wang

**Affiliations:** 1 School of Public Policy, Xiamen University, Xiamen, China; 2 College of Public Management, Guizhou University of Finance and Economics, Guiyang, China; 3 China Academy for Rural Development, Zhejiang University, Hangzhou, China; 4 School of Public Affairs, Zhejiang University, Hangzhou, China; National University of Distance Education: Universidad Nacional de Educacion a Distancia, SPAIN

## Abstract

Based on China Family Panel Studies (CFPS) data from 2012 to 2020, we estimate the effect of the "Universal Two-Child" (UTC) policy on women’s employment income in China by the Difference-in-Difference (DID) model. Our results show that the UTC policy leads to an average decrease of 20.86% in women’s employment income. Moreover, we reveal the mediation effect in the impact of the UTC policy on women’s income and find that the UTC policy leads to a decrease in women’s income by reducing their working hours and hourly wages. Furthermore, we find that the negative impacts of the UTC policy on women’s employment income are greater among women under 35 years old and those without a bachelor’s degree.

## 1. Introduction

Since the 1980s, China’s family planning policies have been evolving and adapting. In 1980, China implemented the strictest "One-Child" policy, which limited all couples to only having one child. In 1984, this policy was slightly relaxed with the introduction of the "One-And-A-Half-Child" policy, which allowed rural couples to have two children if their first child was a girl. These policies were successful in slowing down China’s population growth [1, 2]. By the year 2002, China began facing a labor shortage issue and started to relax the family planning policies, aiming to stimulate population growth. The "One-Child" policy was replaced with the "Two Only-Child Two Children" policy, allowing urban couples to have two children if both of them were the only child in their own families. However, this policy was not as effective in reversing the declining trend in population growth as expected [3]. Subsequently, in 2013, China implemented the "Selective Two-Child" policy, permitting couples to have two children if at least one of them was the only child in their own families. Soon after, the "Universal Two-Child" (UTC) policy was introduced in 2016, allowing couples to have two children if both of them were not the only child in their own families.

As the new family planning policy, the UTC policy has successfully achieved its goal of encouraging a couple to have a second child. According to the data from the National Bureau of Statistics of China, one year after the implementation of the UTC policy, the proportion of the second child in the total population increased by 11% (from 41.2% in 2016 to 51.2% in 2017). Although China’s fertility rate has increased, Chinese women still face some problems in the labor market. According to the World Bank, women’s labor participation in China has continuously declined from 70.52% to 61.82% during the past twenty years, and the gap between men’s and women’s labor participation rates has widened from 11.6% to 14.8% from 1990 to 2019 [4]. Moreover, in addition to a decrease in labor participation rates, women’s employment income is also lower than that of men. Tang and Scott [5] estimated that the gender wage gap in China has reached 25%. These issues are mainly caused by the "motherhood wage penalty", which refers to the childbearing behavior of women can reduce the labor participation of married women, and cause sizable reductions in women’s labor market outcomes [6–10].

Under such circumstances, does the UTC policy cause Chinese women to face a more complicated choice between motherhood and employment? Does it intensify the "motherhood wage penalty" faced by Chinese women? Does it exacerbate gender discrimination in the labor market and harm Chinese women’s labor rights? By conducting empirical research on the impact of the UTC policy on Chinese women’s employment income, and searching for answers to these questions, we may contribute to providing a reference for the Chinese government’s future policy-making, thereby safeguarding women’s labor rights and promoting gender equality.

Currently, many studies in the academic community have evaluated the impacts of China’s "Two-Child" policy. Some studies adopt a pessimistic stance regarding the "Two-Child" policy’s long-term effectiveness in addressing China’s demographic challenges, such as the gender imbalance and the aging population [11–13]. In addition, some studies indicate that the "Two-Child" policy contributes positively to alleviating China’s economic and fiscal pressures [14–16]. In general, existing studies on the "Two-Child" policy impacts tend to focus mainly on the macro-level effects, with limited attention to the influence on micro-level individual behaviors. Even when such studies exist [17], they often overlook the specific effects of the "Two-Child" policy on women’s income in the labor market. Huang and Jin [4] investigated the impact of the "Two-Child" policy on women in urban areas and found that the policy decreased women’s labor income by 10.43%. Indeed, this study is one of the few studies in the existing literature that focuses on the impact of the "Two-Child" policy on women’s income. However, they only used the data up to 2018, while the UTC policy was implemented in 2016, which makes it impossible for them to examine the long-term impact of the UTC policy. Moreover, their regression model had the problem of insufficient control variables, they only controlled the province fixed effect instead of the individual fixed effect or the interaction term of province fixed effect and time fixed effect, which leads to bias caused by omitted variables. Besides, they only superficially estimated the impact of the UTC policy on women’s income, without an in-depth analysis of the mediators by which the UTC policy affects women’s income.

Indeed, extensive studies have investigated the determinants of women’s labor force participation and employment income. For example, Kreps [18] pointed out that the relationship between women’s age and their employment income often presents an inverted U-shaped curve. Semyonov [19] found that women’s labor force participation is positively related to economic development and divorce rate and negatively related to income inequality. Gjerdingen et al. [20] argued that women’s health and labor participation are interdependent, and good health can lead to more women’s participation in labor, while excessive labor can harm women’s health. Moreover, numerous studies have emphasized the significant positive impact of education on women’s labor participation rates [21, 22]. Riordan [23] and Lee et al. [24] highlighted the positive impact of education on women’s ability to increase their own employment income. Similarly, Bobbitt-Zeher [25] also found that women’s gains in education may have been central to narrowing the gender gap in income. The above studies mainly analyze the impact of women’s individual characteristics on their employment income, but they do not consider the influence of fertility behavior on women’s labor force participation.

Many researchers [26–28] have confirmed the existence of the "motherhood wage penalty", they pointed out that women’s fertility behavior has a significant negative impact on their labor participation and salaries. The impact of children on women’s labor market outcomes is widespread, affecting in terms of labor force participation, working hours, and wages [29].

Budig and England [28] argued that childbirth and childcare can lead to interruptions in work experience, less efficient work, choice of mother-friendly jobs rather than highly paid jobs, and discrimination from employers. Herrarte et al. [30] pointed out that having a newborn can have a negative and very significant effect on women’s labor market decisions, and emphasized the male partner’s job characteristics have a relevant effect on women’s employment decisions. Begall and Grunow [31] argued that new mothers in the Netherlands became less likely to exit the labor market and more likely to reduce their working hours from 1970 to 2008. Spiess and Dunkelberg [32] found a significant negative effect of poor maternal mental and physical health on female labor force participation within a year of childbirth. Similarly, Frodermann and Müller [33] found that mothers have lower transition rates to re-employment than childless women. The above literature indicates that childbirth significantly reduces women’s labor force participation and working hours.

Waldfogel [27] found that the hourly wages of women with one child are 6% lower compared to unmarried women, and 13% lower for women with two children. Similarly, based on European data, Davies and Pierre [34] found that compared to women who have not given birth, women who have given birth to one child have a 10% decrease in employment income. Jia and Dong [35] found that mothers earn considerably less than childless women based on Chinese data. Juhn and McCue [36] pointed out that mothers are more inclined to seek part-time jobs or switch their work departments in the process of parenting, and such translations often lead to lower wages. Dumauli [37] examined the effect of the timing of first childbirth on the motherhood wage penalty experienced by working mothers in Japan and found that having children negatively affects the wages of Japanese women but there is no variation in the motherhood wage penalty between early child bearers (age 27 years or younger) and late child bearers (older than 27 years). These studies all indicate that childbirth can lead to a decrease in women’s wages. The above studies mainly focus on the negative impact of micro-level fertility behavior on women’s employment income, however, there is still a need to examine the specific impact of macro-level policies like the UTC policy on women’s income and labor market outcomes, so as to promote women’s economic empowerment in China.

In response to the issues not addressed in the above literature, we apply the Difference-in-Difference model to evaluate the impact of the UTC policy on Chinese women’s labor market outcomes based on the panel data of the China Family Panel Studies from 2012 to 2020. Moreover, we investigate the mediation effect in the UTC policy affecting women’s work income. Finally, we also examine the heterogeneity of the impact of the UTC policy on women’s employment income. In comparison to existing literature, we may mainly contribute in the following parts. On the one hand, we enrich and expand the empirical research on the effects of the UTC policy, focusing on the negative social implications of the UTC policy while it increases China’s fertility rate in the short run [14]. On the other hand, we examine how the UTC policy affects women’s employment income and reveal the different responses of different types of women to the UTC policy. These findings can provide a reference for future policy reforms.

The remainder of the study is organized as follows. Part 2 establishes a theoretical framework and proposes hypotheses. Part 3 establishes the model, which includes the Difference-in-Difference model and the Event Study model. Part 4 introduces the data used in this study, including the source of the data, the definition of the variables and the summary statistical results of the data. Part 5 presents the empirical results, including the estimated results of the baseline regression, the robustness tests, the mediation analysis and the heterogeneity analysis. Part 6 presents the conclusions. Part 7 ends with a discussion.

## 2. Theoretical framework

Many studies [38, 39] have proposed the concept of "motherhood wage penalty". The motherhood wage penalty refers to that mothers are likely to receive lower wages compared to childless women. This observation can be linked to several theories. According to the theory of human capital [26], the motherhood wage gap is caused by differences in the standard human capital characteristics between mothers and non-mothers (such as differences in age and education), as well as the loss and non-accumulation of human capital during employment breaks or reduced working hours related to children [40, 41]. Besides, based on the compensating wage differentials theory [42], mothers are not only likely to work less and have lower work experience, but may also choose different kinds of jobs. One of the reasons for the existence of the motherhood wage penalty is that mothers are more inclined to choose jobs that are more suitable for taking care of children, and the average wages of such jobs are often lower. Furthermore, role conflict theory asserts that motherhood wage penalty is due to the inherent tension between household labor and career development [43]. Women undertake the roles of mothers and employees, which induces virtually exclusive family-work conflict.

In addition, the motherhood wage penalty is also explained by differences in work effort exerted at work by mothers and non-mothers [44]. According to this theoretical approach, mothers invest less energy in their work because they are also engaged in housework and childcare. Moreover, Gough and Noonan [45] proposed that the wage gap between mothers and childless women may also arise due to the selective allocation of women to motherhood. In other words, women who decide to have more or have children earlier may be relatively more inclined towards family rather than career, so they may perform worse in their jobs compared to women without children [46, 47]. Besides the theories listed above, discrimination is also one of the reasons that leads to the motherhood wage penalty. Correll et al. [48] found that mothers are systematically assessed as less competent and are offered lower starting salaries than childless women by an experimental study.

Based on the aforementioned theories, we have established a theoretical framework for the cause of the motherhood wage penalty. Overall, childbirth can lead to a decrease in women’s working hours and hourly wages, resulting in a decrease in their employment income, and numerous studies have also confirmed this opinion [27, 28, 33, 37]. On the one hand, employers may be concerned that women’s work performance could be influenced by childbirth, leading to a decrease in their hiring or retention of female employees; on the other hand, women themselves may reduce their working hours or participation in the labor force actively or passively due to childbirth-related responsibilities. Given that the UTC policy increases the likelihood of women engaging in childbirth behavior, it has also increased the likelihood of women suffering the motherhood wage penalty. Hence, we propose the following hypotheses:

Hypothesis 1: the UTC policy can lead to a significant decrease in women’s employment income.

Hypothesis 2: the UTC policy can lead to a decrease in women’s employment income by reducing their working hours.

Hypothesis 3: the UTC policy can lead to a decrease in women’s employment income by reducing their hourly wages.

Currently, many studies [49–51] indicate that pregnancy after the age of 35 carries a greater health risk. In other words, women older than 35 years have traditionally been termed as of "advanced maternal age", and considered to have a higher incidence of obstetric complications and adverse pregnancy outcomes than younger pregnant women. Hence, women under 35 years old are at a crucial stage in their fertility decisions and typically face significant family planning and childcare responsibilities, making them more receptive to the UTC policy. On the other hand, women over 35 years old usually have already completed the family planning or parenting stage, making it difficult for them to have another child, which means their childbirth decisions are relatively stable. Therefore, the UTC policy may have a relatively smaller impact on their employment income. Based on this theory, we propose the following hypothesis:

Hypothesis 4: the negative impact of the UTC policy on women’s employment income is greater among women under the age of 35.

Lastly, numerous studies have shown that education can significantly increase women’s employment income [23–25]. College-educated women are typically more career-oriented than their non-college educated counterparts and also the group most often observed postponing maternity [52]. Women with higher levels of educational attainment typically have access to better job opportunities, higher earning potential, and greater labor market flexibility [53]. According to this theory, women with higher levels of educational attainment also have more discourse power in whether or not to have children, and the UTC policy has a smaller impact on their childbirth behavior. Hence, we propose the following hypothesis:

Hypothesis 5: the negative impact of the UTC policy on women’s employment income is greater among women without a bachelor’s degree.

## 3. Methods

The Difference-in-Difference (DID) method is widely used in policy evaluation in the academic community [54]. We regard the UTC policy as a quasi-natural experiment and apply the DID method to estimate the impact of the UTC policy on females’ employment income. The UTC policy was implemented in 2016, allowing the BNOC (both members of the couple are not "only child") families to have two children, while other families (at least one member of the couple is "only child") remain unaffected by this policy. We set the following model:

$$Ln{Income}_{it}=\alpha +\beta_{1}{Treat}_{it}+\beta_{2}{Post}_{it}+\beta_{3}{Policy}_{it}+\sum_{j}\theta_{j}{Controls}_{j,it}+\gamma_{t}+\delta_{i}+\gamma_{t}*\epsilon_{i}+\mu_{it}$$ (1)

where Ln*Income*_*it*_ refers to the logarithm of weekly employment income for female *i* at time *t*; *Treat*_*it*_ refers to the dummy variable of whether or not a female is affected by the UTC policy, with a value of "1" if she is in a BNOC family, and "0" otherwise; *Post*_*it*_ refers to the dummy variable of whether or not the UTC policy is implemented, with a value of "1" after 2016 (including 2016), and "0" before 2016; *Policy*_*it*_ is derived by *Treat*_it_*Post_*it*_ and its coefficient (*β*_3_) is the most important coefficient in the model, which represents the average impact of the UTC policy on females’ employment income. Besides, *Controls*_*j*,*it*_ refers to the *j*-th control variables for female *i* at time *t*; *γ*_*t*_ refers to the year fixed effect, which is used to control the factors that only change over the years; *δ*_*i*_ refers to the individual fixed effect, which is used to control the factors that only change over the individuals; *ϵ*_*i*_ refers to the county fixed effect, which is used to control the factors that only change over the counties. The introduction of the interaction term of *γ*_*t*_ and *ϵ*_*i*_ is to control the factors that change over the years at the county level, so as to reduce endogeneity caused by omitted variables. *μ*_*it*_ refers to the error term. In addition, we use the ordinary least square (OLS) method to estimate Eq (1) and adopt the standard errors that are clustered at the individual level to correct the heteroscedasticity problem.

To verify the common trend assumption of the DID specification, we apply the Event Study model to estimate the dynamic effect of the UTC policy on females’ employment income. Following Jia et al. [3] and Han and Wu [55], using the year 2014 (the previous period before policy implementation) as the base year, we set the following model:

$$Ln{Income}_{it}=\alpha +\beta_{1}{Treat}_{it}+\sum_{k}\left(\beta_{k,2}{Period}_{k,it}+\beta_{k,3}{Period}_{k,it}*{Treat}_{it}\right)+\sum_{j}\theta_{j}{Controls}_{j,it}+\gamma_{t}+\delta_{i}+\gamma_{t}*\epsilon_{i}+\mu_{it}$$ (2)

where *Period*_*k*,*it*_ refers to the dummy variable of whether or not a female is in the *k*-th period (*k* ranges from 1 to 4). For instance, the value of *Period*_1,*it*_ is "1" if the year is 2012, and "0" otherwise; the value of *Period*_2,*it*_ is "1" if the year is 2016, and "0" otherwise; the value of *Period*_3,*it*_ is "1" if the year is 2018, and "0" otherwise; the value of *Period*_4,*it*_ is "1" if the year is 2020, and "0" otherwise. The other variables in Eq (2) are the same as in Eq (1). All participants or their legal representatives in the China Family Panel Studies have signed written informed consent forms to participate in the baseline and follow-up surveys. Informed consent was obtained from study participants before completing the study questionnaire. Our study was approved by the Biomedical Ethics Committee of Peking University (IRB00001052-11015).

## 4. Data

Data used in this study is obtained from the China Family Panel Studies (CFPS), which is launched by Peking University, and is a nearly nationwide, comprehensive, longitudinal social survey that is intended to serve research needs on a large variety of social phenomena in contemporary China. At present, the CFPS database contains a total of six waves of data (2010, 2012, 2014, 2016, 2018, 2020). However, due to the small sample size of 2010 data and significant differences in questionnaire design compared to the later waves, this study opts to utilize data from five waves after 2012 (i.e., 2012, 2014, 2016, 2018, 2020) as the sample for analysis.

We clean the raw data via the following procedures. Firstly, we retain only the female samples with a marital status of "married" from the years 2012 to 2020. Secondly, samples of females aged above 49 years are excluded because their child-bearing is very rare. Lastly, samples of females who had more than one child before the policy implementation are removed, as they are not affected by the UTC policy anyway. The final sample size for the study is 10,225, of which 5,637 are from the BNOC families and 4,588 are from other families.

The dependent variable of this study is expressed as the females’ weekly employment income (adjusted by the consumer price index). The independent variable of this study is "Policy" in Eq (1). Following Kreps [18], Gjerdingen [20] and Riordan [23], we choose control variables in several categories. First are female characteristic variables, including age, the square of age, educational attainment, health status and registered permanent residence. Second are household characteristic variables, including per capita family net income (adjusted by the consumer price index) and per capita family asset (adjusted by the consumer price index). Third are first child characteristic variables, including age and gender. In addition, females’ weekly working hours and hourly wages are introduced in this study as mediators. Summary statistics for the main variables are shown in Table 1.

**Table 1:** Summary statistics.

*Var*.	Definition (Unit)	Obs.	Mean	S. D.	Min	Max
*Dependent variable*						
Income	Weekly income through work (Yuan)	2,645	462.73	178.91	152.07	868.75
*Control variables*						
Age	Age (Year)	10,225	33.92	7.35	16	49
Education	Years of education (Year)	10,225	9.26	4.36	0	16
Health	Self-rated health level, Unhealthy = 1, Average = 2, Healthy = 3	10,225	2.72	0.59	1	3
Urban	Registered permanent residence, Urban = 1, Rural = 0	10,225	0.26	0.44	0	1
AverageFI	Per capita family income (Thousand Yuan)	10,225	17.85	15.23	0.64	121.97
AverageFA	Per capita family asset (Thousand Yuan)	10,225	126.68	188.10	1.79	1,696.47
GenderFC	Gender of the first child, Male = 1, Female = 0	10,225	0.55	0.50	0	1
AgeFC	Age of the first child (Year)	10,225	9.73	7.32	0	34
*Mediators*						
Time	Weekly working hours (Hours)	4,166	47.24	9.04	29.00	66.50
Wage	Hourly wages (Yuan/Hour)	2,123	10.21	4.40	2.72	23.08

*Notes*: "Income", "AverageFI" and "AverageFA" are adjusted by the consumer price index. "Wage" is calculated by "Income" / "Time". Because there are a large number of samples that are not employed, the sample sizes of "Income" and "Time" are much smaller than that of other variables.

Based on Table 1, it can be observed that the women have an average education duration of 9 years, meeting China’s compulsory education standard, although some women have an education duration of 0, indicating they have not received formal education. The average self-rated health level for women is 2.72, which is very close to 3, implying that the vast majority of women consider themselves to be in good health. In addition, it is worth noting that a significant number of samples are not employed, resulting in a much smaller sample size for "Income" and "Time" in Table 1 compared to other variables. Out of the total 10,225 samples, 5,537 samples are not employed, leaving only 4,688 samples with jobs. Among these employed samples, 2,043 have missing "Income", and 522 have missing "Time".

Lastly, given that the validity of DID estimates is predicated on that there should be no systematic difference between the women affected by the UTC policy and the women not affected by the UTC policy. Referring to Jia et al. [3], we conduct a mean t-test on control variables between these two groups, results are shown in Table 2. Similar to the observations of Jia et al. [3], we also find that there is a significant statistical difference in the mean of these two groups. Considering that our study is not a randomized controlled trial (RCT), we are unable to ensure that these two groups are assigned randomly, which explains why there is a significant statistical difference in the mean of these two groups and the importance of including these variables in the regression model. Furthermore, although there is a significant statistical difference in the mean of these two groups, the value of the difference is relatively small. For instance, the difference in Education is 0.18, while the mean of the whole sample is 9.26, which means that the difference is only 1.9% of the mean of the whole sample. This indicates that the women affected by the UTC policy and the women not affected by the UTC policy in our data are comparable.

**Table 2:** Mean t-test of control variables.

*Var*.	Unaffected group		Affected group		Difference
	Obs.	Mean	Obs.	Mean	
Age	4,588	35.28	5,637	32.80	2.48***
Education	4,588	9.36	5,637	9.18	0.18**
Health	4,588	2.68	5,637	2.75	-0.07***
Urban	4,588	0.33	5,637	0.21	0.12***
GenderFC	4,588	0.56	5,637	0.54	0.02**
AgeFC	4,588	10.85	5,637	8.82	2.03***
AverageFI	4,588	18.12	5,637	17.64	0.48*
AverageFA	4,588	142.28	5,637	113.99	28.29***

*Notes*:

***, ** and * denote statistical significance, at the 1%, 5%, and 10% levels, respectively. "Difference" is computed by: Mean of Unaffected group minus Mean of Affected group.

## 5. Empirical results

### 5.1. Impact of the UTC policy on women’s income

Table 3 reports the regression results on the impact of the UTC policy on females’ employment income. In column (1), no control variables or fixed effects are included; in column (2), year fixed effect and individual fixed effect are added; in column (3), the interaction term of year fixed effect and county fixed effect is introduced; in column (4), female characteristics variables are added as control variables; in column (5), household characteristics variables are included as control variables; in column (6), first-born child characteristics variables are introduced as control variables.

**Table 3:** Impact of the UTC policy on women’s income.

*Dep Var*.	Ln(Income)					
	(1)	(2)	(3)	(4)	(5)	(6)
Treat	0.0125	0.0687	0.1228	0.1420	0.1420	0.1375
	(0.0332)	(0.0585)	(0.0863)	(0.0896)	(0.0907)	(0.0860)
Post	0.1309***					
	(0.0252)					
Policy	-0.0626	-0.0988*	-0.2012**	-0.2052**	-0.2073**	-0.2086***
	(0.0390)	(0.0568)	(0.0803)	(0.0818)	(0.0833)	(0.0789)
Age				0.0267	0.0262	0.0265
				(0.0520)	(0.0521)	(0.0502)
Age^2^				-0.0004	-0.0003	-0.0002
				(0.0006)	(0.0006)	(0.0006)
Education				0.0262**	0.0260*	0.0255**
				(0.0133)	(0.0133)	(0.0128)
Health				-0.0251	-0.0246	-0.0313
				(0.0328)	(0.0331)	(0.0316)
Urban				-0.1006	-0.0986	-0.1036
				(0.0652)	(0.0645)	(0.0665)
GenderFC					0.0509	0.0741
					(0.1278)	(0.1233)
AgeFC					0.0060	0.0041
					(0.0091)	(0.0090)
Ln(AverageFI)						0.0978***
						(0.0338)
Ln(AverageFA)						-0.0233
						(0.0225)
Intercept	5.9852***	6.0931***	6.1113***	5.4494***	5.3551***	4.4761***
	(0.0190)	(0.0169)	(0.0250)	(1.4625)	(1.4677)	(1.4780)
Year *FE*	NO	YES	YES	YES	YES	YES
Individual *FE*	NO	YES	YES	YES	YES	YES
County-Year *FE*	NO	NO	YES	YES	YES	YES
Obs.	2,645	2,645	2,645	2,645	2,645	2,645
R-squared	0.0134	0.7169	0.8466	0.8500	0.8501	0.8537

*Notes*: Standard errors are clustered at the individual level and presented in parentheses.

***, ** and * denote statistical significance, at the 1%, 5%, and 10% levels, respectively. "Post" in columns (2)—(6) is absorbed by fixed effects.

We take the regression in column (6) as the baseline regression. Based on the results of the regression in column (6), results show that the coefficient of "Policy" is -0.2086, and it is statistically significant at the 1% level. This indicates that the UTC policy has a significant negative impact on women’s employment income, leading to an average decrease of 20.86% in their earnings. Descriptive statistics show that the average weekly work income of women is 463 yuan, indicating that the UTC policy has led to an average decrease of 97 yuan in women’s weekly employment income.

Furthermore, in columns (1) to (3), the gradual inclusion of fixed effects results in the coefficient of "Policy" changing from statistically insignificant to significant, and the R-squared of the model progressively increases to 85%. This implies that the fixed effects controlled in this study are comprehensive, effectively addressing endogeneity bias caused by omitted variables.

Moreover, in columns (4) to (6), with the gradual addition of control variables, the "Policy" coefficient maintains the significance without significant variation. This implies that the independent variable in this study is nearly exogenous, not influenced by the various characteristic variables included as control variables. Hence, the baseline regression in this study is robust and compelling.

**Fig 1:**
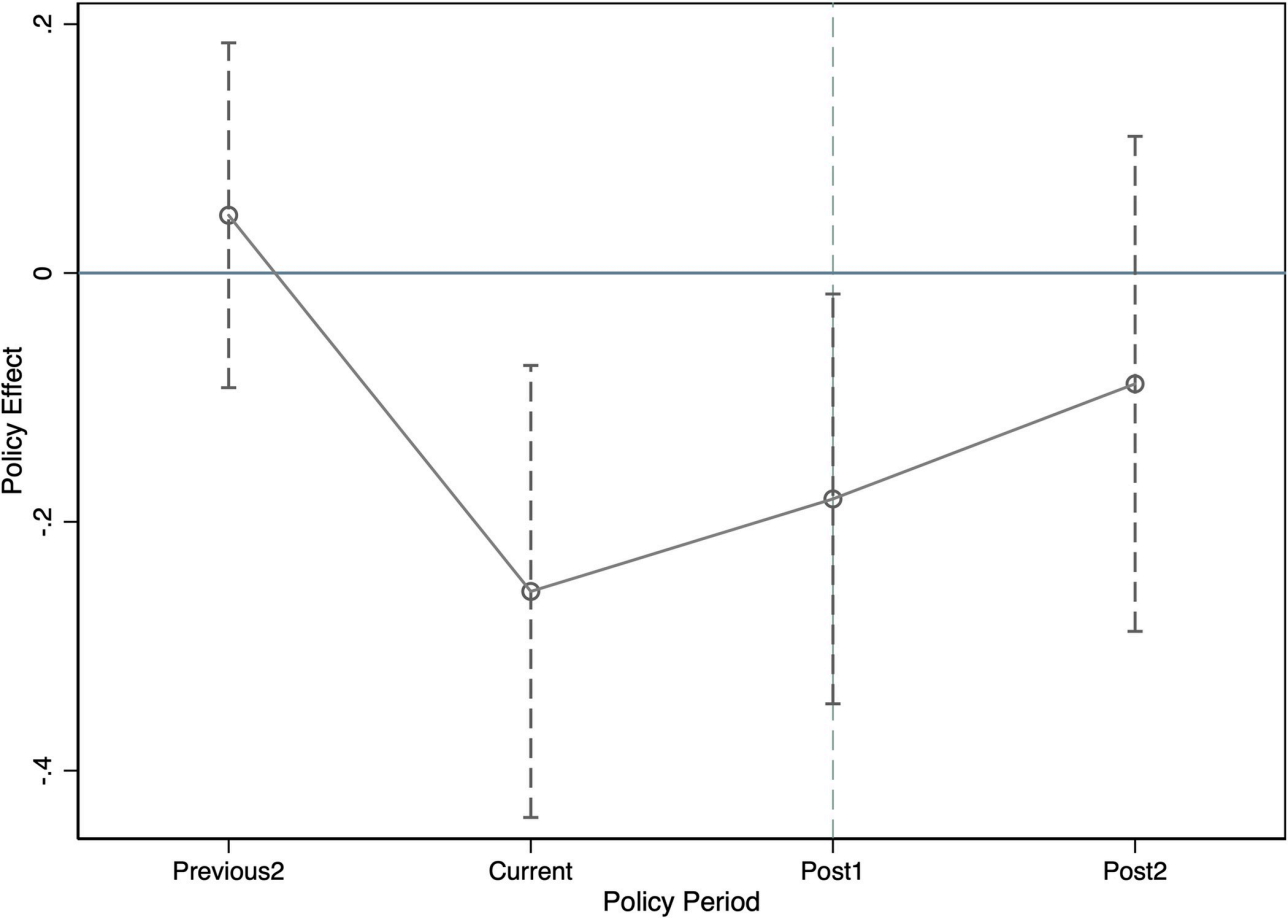
Common trend test. *Notes*: Taking the previous period before the policy (2014) as a comparison, "Previous2" corresponds to 2012, "Current" corresponds to 2016, "Post1" corresponds to 2018, and "Post2" corresponds to 2020.

In addition, the validity of DID estimates is predicated on meeting the common trend hypothesis prior to the policy. Therefore, we utilize the event study method, referring to Eq (2), to conduct a common trend test on the baseline regression. The results of the common trend test are presented in Table 4 and Fig 1.

**Table 4:** Common trend test.

*Dep Var*.	Ln(Income)
	(1)
Previous2*Treat	0.0464
	(0.0841)
Current*Treat	-0.2560**
	(0.1102)
Post1*Treat	-0.1816*
	(0.0999)
Post2*Treat	-0.0891
	(0.1207)
Treat	0.1091
	(0.1015)
Previous2	
Current	
Post1	
Post2	
Intercept	4.5580***
	(1.4761)
Control *Vars*.	YES
Year *FE*	YES
Individual *FE*	YES
County-Year *FE*	YES
Obs.	2,645
R-squared	0.8547

*Notes*: Standard errors are clustered at the individual level and presented in parentheses.

***, ** and * denote statistical significance, at the 1%, 5%, and 10% levels, respectively. "Previous2" represents the 2012 dummy variable. "Current" represents the 2016 dummy variable. "Post1" represents the 2018 dummy variable. "Post2" represents the 2020 dummy variable. "Preivous2", "Current", "Post1" and "Post2" are absorbed by fixed effects.

Based on Table 4, when using the previous period before policy implementation (2014) as the baseline year, the coefficient of "Previous2" (2012) is not statistically significant, while the coefficient of "Current" (2016) is negative and significant at the 1% level. This indicates that before the policy implementation, there was no significant difference in the trend of changes in employment income between the women affected by the UTC policy and the women not affected by the UTC policy. However, after the policy implementation, there was a significant difference in the trend of changes in employment income between the two groups, with the women affected by the UTC policy experiencing a significant decrease of 25.6% in employment income compared to the women not affected by the UTC policy. Additionally, the coefficient of "Post1" (2018) is negative and still significant at the 10% level, while the coefficient of "Post2" (2020) is not statistically significant. This suggests that the negative impact of the UTC policy on women’s employment income persisted until 2018, and it only became statistically insignificant by the year 2020. Based on Fig 1, it is evident that the 90% confidence interval for "Previous2" encompasses "0", while the 90% confidence interval for "Current" does not include "0". In conclusion, the baseline regression in this study meets the common trend hypothesis, and its results are deemed valid.

### 5.2. Robustness tests

**Fig 2:**
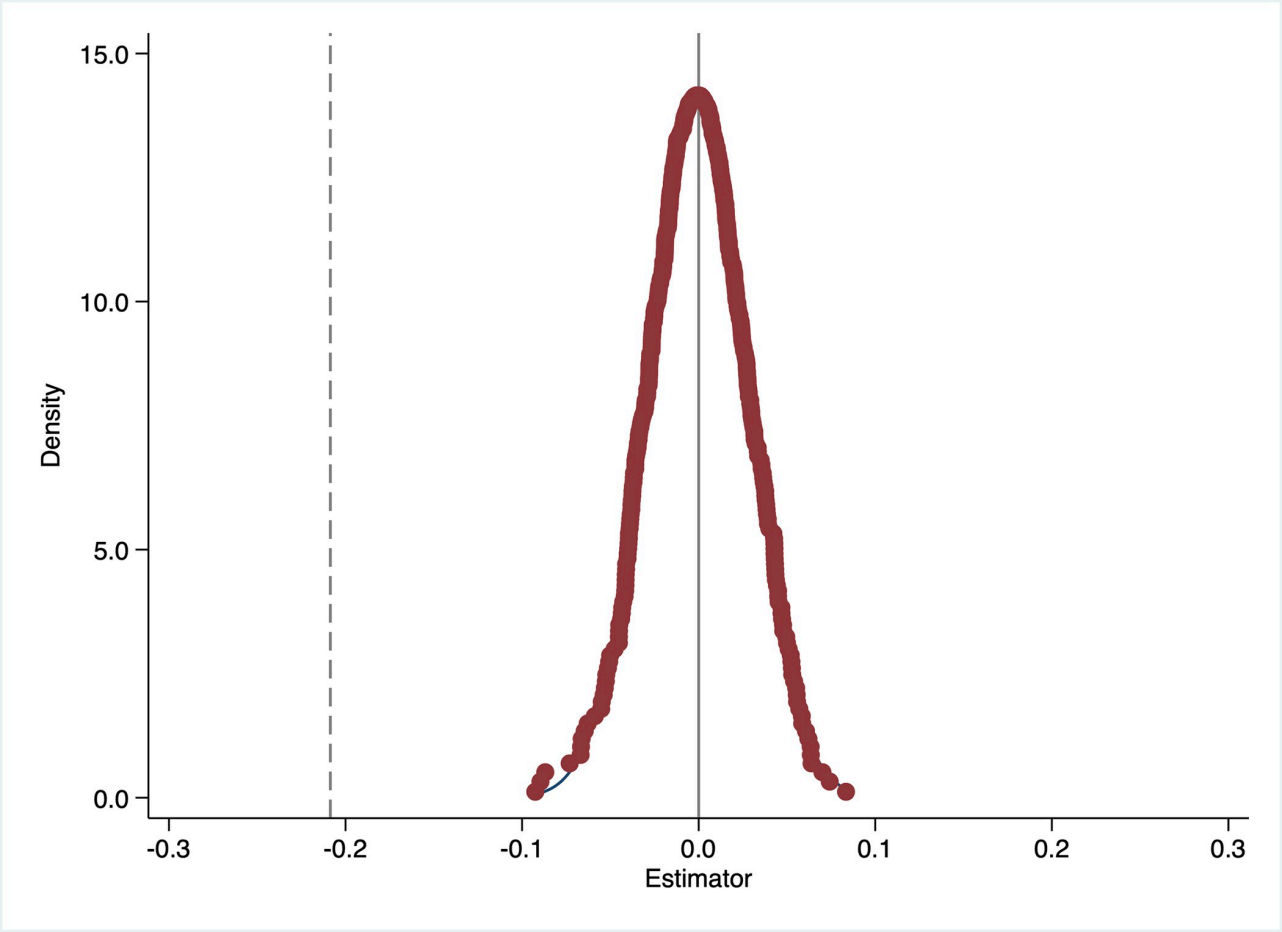
Placebo test. *Notes*: The dashed line corresponds to the estimated policy impact in the baseline regression.

To further validate the robustness of the baseline regression in this study, a placebo test is conducted by making the policy shock to females to be random [56]. Specifically, we randomly set a group of females to be affected by the UTC policy (randomly generated by computer), then construct a false "Treat" based on the false group, while "Post" still refers to policy implementation in 2016, then we re-estimate Eq (1), and we are still most interested with the coefficient of "Policy". To make the placebo test more convincing, we repeat this exercise 500 times. If the change in women’s employment income is indeed due to the UTC policy, then we should find that most of these "Policy" coefficients have values close to "0" and no significant results in the placebo test, because these groups are false and not actually affected. Fig 2 shows the distribution of the coefficients of "Policy" estimated after the random draws. From Fig 2 we can observe that the estimation coefficients of "Policy" are very close to zero, indicating that there are no policy effects for the group randomly assigned. Hence, the placebo test results largely support the baseline regression results that the UTC policy indeed causes negative changes in women’s employment income.

In addition, given the substantial presence of non-employed females within our sample, only those who are employed have the opportunity to obtain employment income, while the non-employed females lack any such earning prospects. Consequently, the baseline regression in this study may be susceptible to sample self-selection issues, potentially leading to sample selection bias. To address this concern, we apply the Heckman Two-Step Estimation model [57] to re-evaluate the impact of the UTC policy on women’s employment income. In the first step of the Heckman Two-Step Estimation method, we set a Probit model to capture the factors influencing the probability of women being employed:

$$Prob(z_{it}=1|\omega )=\Phi (\omega \gamma )$$ (3)

where *Prob*(*z*_*it*_ = 1|*ω*) refers to the employment probability for female *i* at time *t*; *ω* refers to a vector of the control variables that may affect the employment probability, in addition to the control variables included in Eq (1), *ω* also includes the number of children as constraint variable. *γ* refers to a vector of the coefficients. Based on Eq (3), we can calculate the Inverse Mills Ratio (IMR) and add it as a correction term for selection bias into Eq (1), then we re-estimate Eq (1). We expect to see that the IMR is not significant in the new estimation results, indicating that our original baseline regression is not affected by the sample self-selection problem and there is no selection bias.

Besides, considering that there may still be possible unobservable and unchangeable intergroup differences between the women affected by the UTC policy and the women not affected by the UTC policy, we further use the propensity score matching (PSM)-DID model proposed by Heckman et al. [58] to address this concern. The PSM can find the most similar samples in the women affected by the UTC policy and the women not affected by the UTC policy for comparison; after matching the samples, both groups do not differ significantly in the control variables. We utilize the control variables from Eq (1) as the characteristic variables and employ Logit estimation with a 1:4 nearest neighbor matching approach for the PSM. Subsequently, we re-estimate the Eq (1) using the matched sample. Our expectation is to observe that the coefficient of "Policy" remains statistically significant and negative in the new estimation results.

Finally, to verify the robustness of the standard errors that clustered at the individual level, we further adopt the ordinary standard errors and the robust standard errors to re-estimate Eq (1). We expect to see that the coefficient of "Policy" maintains statistically significant and negative in the new estimation results.

The results of robustness tests are represented in Table 5. Column (1) shows the estimation results of the Heckman Two-Step Estimation model. The coefficient of "IMR" is insignificant and the coefficient of "Policy" is negative and significant at the 5% level. This indicates that our baseline regression is not affected by the self-selection problem and there is no selection bias. Column (2) displays the estimation results of the PSM-DID model. The coefficient of "Policy" is also negative and significant at the 5% level. This means that our baseline regression is robust and not affected by the possible unobservable and unchangeable intergroup differences between the women affected by the UTC policy and the women not affected by the UTC policy. Columns (3) and (4) show the estimation results of regressions adopting different standard errors. The coefficients of "Policy" are both negative and significant at the 5% level in two regressions. This suggests that the significance of the coefficient of "Policy" is robust and it is not affected by the different adoptions of standard errors.

**Table 5:** Robustness tests.

*Dep*. *Var*.	Ln(Income)			
	Heckman	PSM-DID	Ordinary S. E.	Robust S. E.
	(1)	(2)	(3)	(4)
Treat	0.1840*	0.1236	0.1375	0.1375
	(0.1069)	(0.0939)	(0.0901)	(0.0948)
Post				
Policy	-0.2729**	-0.1738**	-0.2086**	-0.2086**
	(0.1212)	(0.0838)	(0.0849)	(0.0884)
IMR	-0.4037			
	(0.5660)			
Age	-0.0003	0.0144	0.0265	0.0265
	(0.0612)	(0.0946)	(0.0714)	(0.0576)
Age^2^	0.0002	0.0002	-0.0002	-0.0002
	(0.0007)	(0.0005)	(0.0005)	(0.0006)
Education	0.0164	0.0289**	0.0255*	0.0255*
	(0.0174)	(0.0132)	(0.0135)	(0.0140)
Health	-0.0435	-0.0026	-0.0313	-0.0313
	(0.0357)	(0.0356)	(0.0348)	(0.0340)
Urban	-0.1393*	-0.1309	-0.1036	-0.1036
	(0.0844)	(0.0801)	(0.0712)	(0.0740)
GenderFC	0.0602	0.1302	0.0741	0.0741
	(0.1224)	(0.1534)	(0.1427)	(0.1403)
AgeFC	-0.0004	-0.0030	0.0041	0.0041
	(0.0114)	(0.0092)	(0.0154)	(0.0110)
Ln(AverageFI)	0.0414	0.1017***	0.0978***	0.0978***
	(0.0903)	(0.0354)	(0.0353)	(0.0364)
Ln(AverageFA)	-0.0109	-0.0469*	-0.0233	-0.0233
	(0.0306)	(0.0243)	(0.0226)	(0.0239)
Intercept	5.7947**	4.6023	4.4761*	4.4761**
	(2.3491)	(3.0637)	(2.3572)	(1.7796)
Year *FE*	YES	YES	YES	YES
Individual *FE*	YES	YES	YES	YES
County-Year *FE*	YES	YES	YES	YES
Obs.	2,506	2,452	2,645	2,645
R-squared	0.8536	0.8599	0.8537	0.8537

*Notes*: Standard errors are presented in parentheses.

***, ** and * denote statistical significance, at the 1%, 5%, and 10% levels, respectively. Column (1) displays the estimation results of the Heckman two-step model. Column (2) displays the estimation results of the PSM-DID model. The standard error used in columns (1)—(2) are clustered at the individual level. The ordinary standard error is adopted in column (3). The robust standard error is adopted in column (4). "Post" in columns (1)—(4) is absorbed by fixed effects. "IMR" represents the Inverse Mills Ratio.

### 5.3. Mediation analysis

In the baseline regression, we have confirmed that the UTC policy has a significant negative impact on women’s employment income. In this section, we further investigate the mediators by which the UTC policy affects women’s employment income. Women’s employment income consists of two parts: one is their working hours, and the other is their wages. Hence, the UTC policy may lead to a decrease in women’s employment income by reducing their working hours or wages. To verify this hypothesis, we set the following model:

$$Ln{Time}_{it}=\alpha +\beta_{1}{Treat}_{it}+\beta_{2}{Post}_{it}+\beta_{3}{Policy}_{it}+\sum_{j}\theta_{j}{Controls}_{j,it}+\gamma_{t}+\delta_{i}+\gamma_{t}*\epsilon_{i}+\mu_{it}$$ (4)

$$Ln{Income}_{it}=\alpha +\beta_{1}{Treat}_{it}+\beta_{2}{Post}_{it}+\beta_{3}{Policy}_{it}+\beta_{4}Ln{Time}_{it}+\sum_{j}\theta_{j}{Controls}_{j,it}+\gamma_{t}+\delta_{i}+\gamma_{t}*\epsilon_{i}+\mu_{it}$$ (5)

where Ln*Time*_*it*_ refers to the logarithm of weekly working hours for female *i* at time *t*; the other variables in Eqs (4) and (5) are the same as in Eq (1). Besides, Ln*Time*_*it*_ can be replaced by Ln*Wage*_*it*_ which refers to the logarithm of wages for female *i* at time *t*. We are most interested in the coefficient of "Policy" in Eqs (4) and (5), and the coefficient of "LnTime" in Eq (5). We expect to see the *β*_3_ in Eqs (4) and (5) to be negative and significant and the *β*_4_ in Eq (5) to be positive and significant. This implies that our hypothesis about the mediators is valid.

We estimate Eqs (4) and (5), the results are represented in Table 6. Column (1) uses the weekly working hours as the dependent variable; column (2) adds the weekly working hours as the mediator. Similarly, column (3) uses the wage as the dependent variable; column (4) adds the wage as the mediator. In column (1), the coefficient of "Policy" is -0.0602 and significant at the 5% level. This means that the UTC policy has resulted in a 6.02% decrease in women’s working hours. In column (3), the coefficient of "Policy" is -0.1792 and significant at the 10% level. This indicates that the UTC policy has caused a 17.92% reduction in women’s wages.

**Table 6:** Mediation analysis.

*Dep*. *Var*.	Ln(Time)	Ln(Income)	Ln(Wage)	Ln(Income)
	(1)	(2)	(3)	(4)
Treat	0.0681**	0.0232	-0.0381	0.0906*
	(0.0342)	(0.1195)	(0.1253)	(0.0508)
Post				
Policy	-0.0602**	-0.2154**	-0.1792*	-0.0883*
	(0.0306)	(0.0977)	(0.0998)	(0.0458)
Ln(Time)		0.3690**		
		(0.1453)		
Ln(Wage)				0.8276***
				(0.0382)
Age	0.0583*	0.0141	0.0278	-0.0169
	(0.0344)	(0.0524)	(0.0518)	(0.0269)
Age^2^	-0.0003	0.0002	0.0001	0.0002
	(0.0002)	(0.0007)	(0.0007)	(0.0004)
Education	-0.0079*	0.0056	0.0112	-0.0069
	(0.0047)	(0.0151)	(0.0170)	(0.0071)
Health	-0.0085	-0.0194	0.0109	-0.0461**
	(0.0159)	(0.0424)	(0.0460)	(0.0216)
Urban	0.0072	-0.0246	-0.0008	-0.0378
	(0.0300)	(0.0738)	(0.0736)	(0.0416)
GenderFC	0.0408	0.0293	-0.0116	0.0627
	(0.0452)	(0.2718)	(0.2535)	(0.0919)
AgeFC	-0.0068	0.0194	0.0124	0.0132
	(0.0061)	(0.0154)	(0.0134)	(0.0088)
Ln(AverageFI)	0.0208	0.0798*	0.0740	0.0219
	(0.0140)	(0.0412)	(0.0450)	(0.0215)
Ln(AverageFA)	-0.0010	-0.0522**	-0.0406*	-0.0253*
	(0.0091)	(0.0234)	(0.0245)	(0.0139)
Intercept	2.1901*	3.6086**	0.7639	4.6400***
	(1.1230)	(1.6268)	(1.3451)	(0.7107)
Year *FE*	YES	YES	YES	YES
Individual *FE*	YES	YES	YES	YES
County-Year *FE*	YES	YES	YES	YES
Obs.	4,166	2,123	2,123	2,123
R-squared	0.7287	0.8721	0.8908	0.9651

*Notes*: Standard errors are clustered at the individual level and presented in parentheses.

***, ** and * denote statistical significance, at the 1%, 5%, and 10% levels, respectively. "Post" in columns (1)—(4) is absorbed by fixed effects.

Besides, the coefficients of "Policy" in columns (2) and (4) are significantly negative, while the coefficient of "LnTime" in column (2) and the coefficient of "LnWage" in column (4) are both significantly positive. Hence, the hypothesis mentioned above in this study is valid, the UTC policy has resulted in a reduction in both the working hours and wages of women, consequently leading to a decline in their overall employment income.

### 5.4. Heterogeneity analysis

In this section, we conduct a heterogeneity analysis to explore whether the negative impact of the UTC policy on women’s employment income varies across different sample subgroups. This heterogeneity analysis sheds light on how certain groups of women may be more vulnerable to the negative consequences of the UTC policy. Understanding such heterogeneity is essential for designing targeted policies that address the specific needs and challenges faced by different groups of women.

Firstly, we divide the sample into two groups based on whether the women are above or below the appropriate childbearing age (35 years old). One group includes women whose age is below 35 years, while the other group comprises women whose age is equal to or above 35 years. This division allows us to examine whether the negative impact of the UTC policy on women’s employment income differs between these two age groups. On the one hand, women below the appropriate childbearing age are at a crucial stage in their fertility decisions and typically face significant family planning and childcare responsibilities, making them more receptive to policy changes. On the other hand, women above the appropriate childbearing age have usually completed their family planning or childcare phases, and hard to have another child, which means that their fertility decisions are relatively stable. As a result, policy changes may have a lesser impact on their employment income. In general, it is expected that women below the appropriate childbearing age are more likely to be affected by the UTC policy, while women above the appropriate childbearing age are relatively less sensitive to the impact of the UTC policy.

Secondly, we classify the sample into two groups based on whether the women are with or without a bachelor’s degree. One group consists of women with less than 16 years of education, and the other group comprises those with 16 or more years of education. This division allows us to examine whether the UTC policy’s impact on women’s employment income differs between women with different educational backgrounds. Women with higher levels of educational attainment typically have access to better job opportunities, higher earning potential, and greater labor market flexibility. Hence, we infer that women with advanced education may experience a relatively smaller negative impact from the UTC policy.

**Fig 3:**
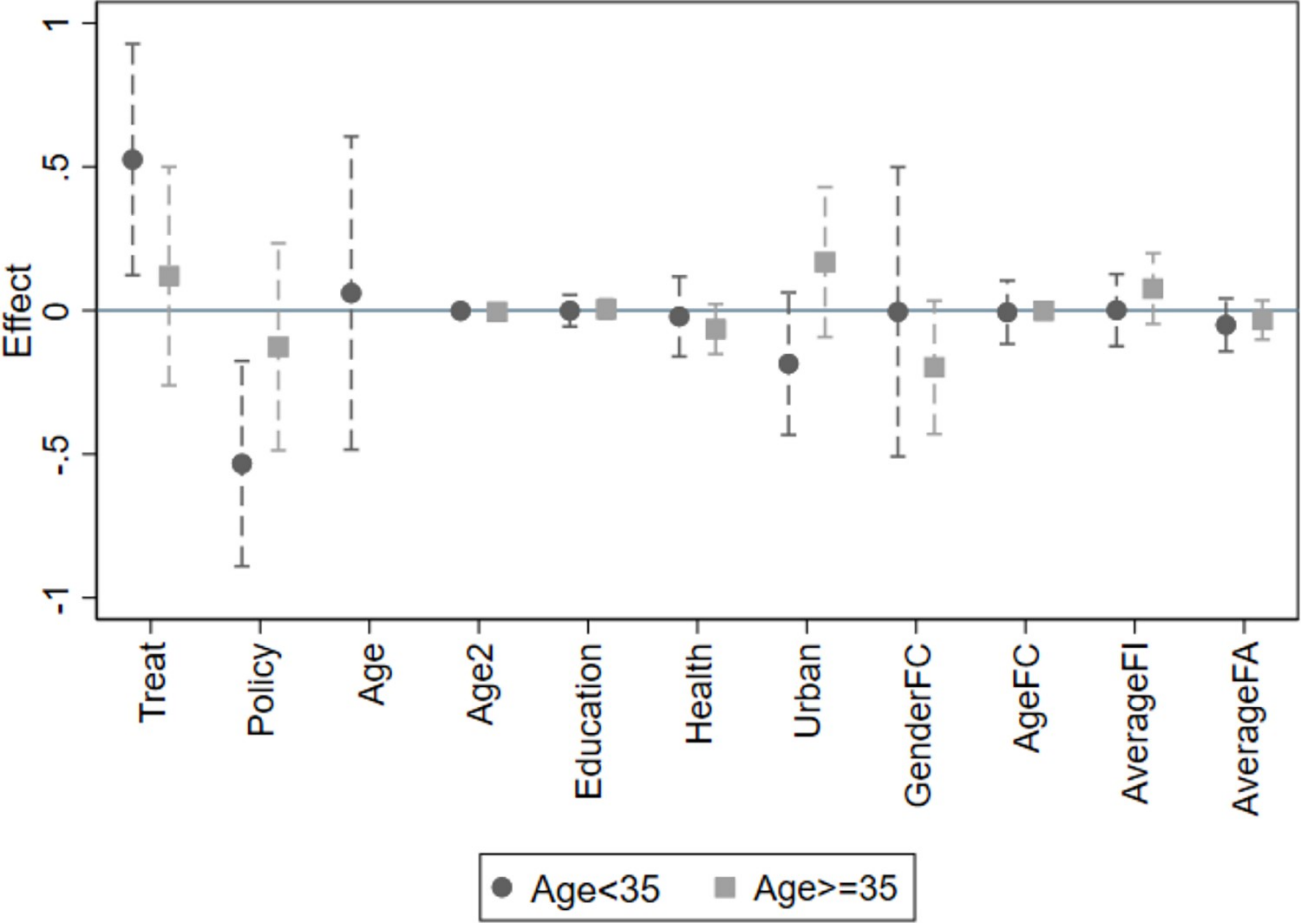
Heterogeneity analysis (Age group). Notes: The fig plots point estimates and 90% confidence intervals for the effect of variables on women’s employment income.

**Fig 4:**
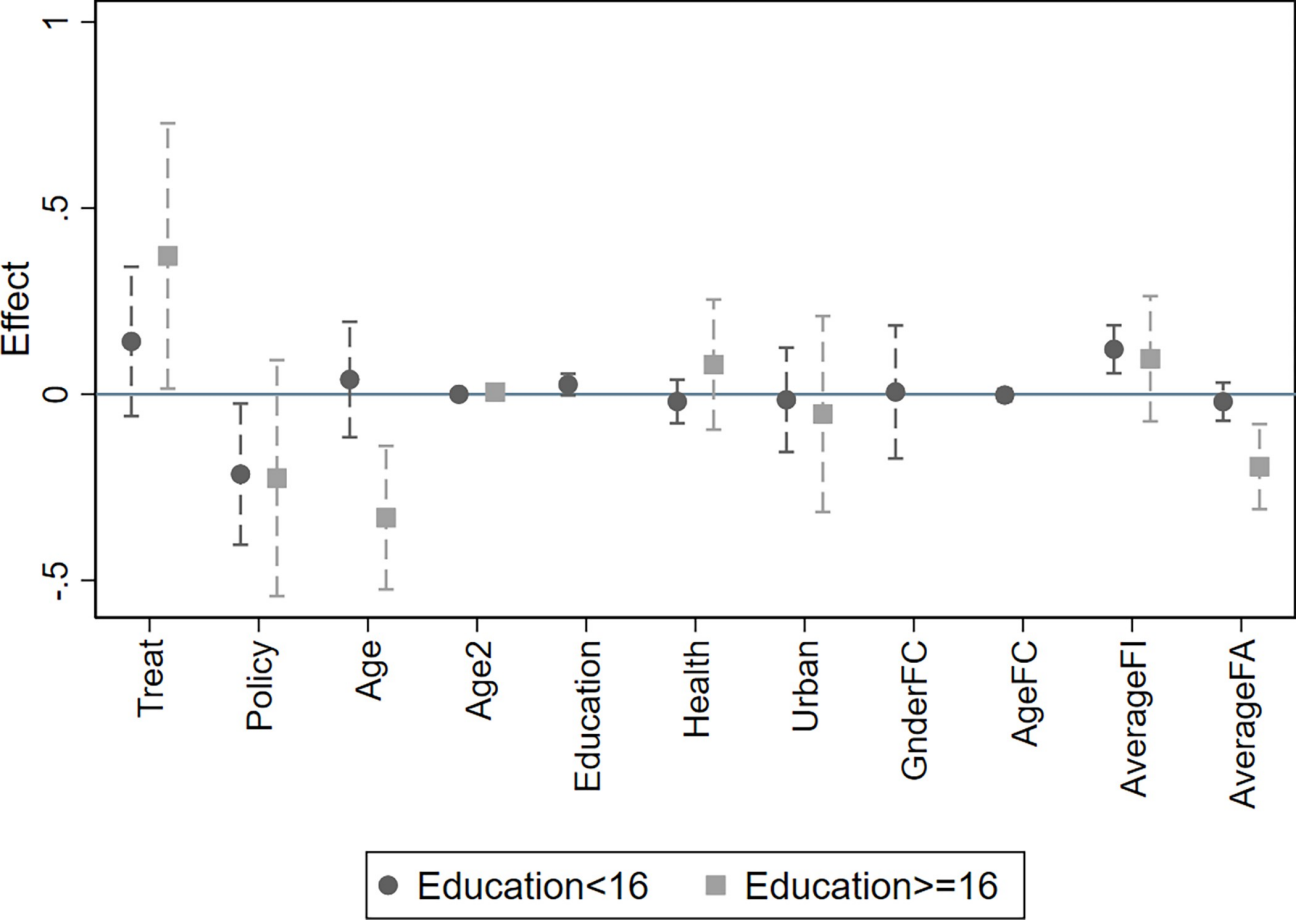
Heterogeneity analysis (Education group). Notes: The fig plots point estimates and 90% confidence intervals for the effect of variables on women’s employment income.

To validate the aforementioned hypotheses, we conduct regression analyses on various subgroups within the sample, and the results are presented in Table 7, Figs 3 and 4. The regression in column (1) only uses the samples younger than 35 years old; while the regression in column (2) only uses the samples that are 35 years or older. The coefficient of "Policy" in column (1) is significantly negative and the coefficient of "Policy" in column (2) is not significant. This indicates that the UTC policy’s negative impact on employment income is statistically significant only for women within the appropriate childbearing age, while it does not have a significant effect on the employment income of women who are above the appropriate childbearing age. Similarly, From the results in column (3), we can see that the coefficient of "35Age*Policy" is -0.2444 and significant at the 5% level, which means that compared to women above 35 years old, the UTC policy resulted in a 24.44% decrease in the employment income of women below 35 years old. This result aligns with our earlier hypothesis, suggesting that women within the appropriate childbearing age are more susceptible to the UTC policy’s consequences due to their active engagement in family planning and childcare responsibilities.

**Table 7:** Heterogeneity analysis.

*Dep*. *Var*.	Ln(Income)					
	Age<35	Age> = 35	Whole Sample	Education<16	Education> = 16	Whole Sample
	(1)	(2)	(3)	(4)	(5)	(6)
Treat	0.5253**	0.1194	0.1252	0.1419	0.3716*	0.1776*
	(0.2429)	(0.2297)	(0.0943)	(0.1215)	(0.2127)	(0.0987)
Post						
Policy	-0.5330**	-0.1266	-0.1825**	-0.2146*	-0.2252	-0.2860***
	(0.2154)	(0.2177)	(0.0907)	(0.1149)	(0.1891)	(0.0887)
35Age*Policy			-0.2444**			
			(0.1100)			
Bachelor*Policy						-0.3759**
						(0.1751)
35Age			0.0120			
			(0.0725)			
Bachelor						0.0128
						(0.1541)
Intercept	6.2584	14.9702***	4.5696**	3.4690	11.5979***	0.0268
	(5.8213)	(3.4531)	(2.1352)	(2.9140)	(2.9188)	(1.7061)
Control Variables	YES	YES	YES	YES	YES	YES
Year *FE*	YES	YES	YES	YES	YES	YES
Individual *FE*	YES	YES	YES	YES	YES	YES
County-Year *FE*	YES	YES	YES	YES	YES	YES
Obs.	1,491	1,154	2.645	1,972	673	2.645
R-squared	0.8919	0.8810	0.8511	0.8520	0.9268	0.8507

*Notes*: Standard errors are clustered at the individual level and presented in parentheses.

***, ** and * denote statistical significance, at the 1%, 5%, and 10% levels, respectively. Due to space constraints, the coefficients of control variables have not been reported. Control variables are the same as in Table 3. "Post" in columns (1)—(6) are absorbed by fixed effects. Samples used in column (1) are less than 35 years old, while samples used in column (2) are 35 years or older. Samples used in column (4) are without a bachelor’s degree, while samples used in column (5) are with a bachelor’s degree or above. "35Age" represents the dummy variable of whether a female is below 35 years old, yes = 1, no = 0. "Bachelor" represents the dummy variable of whether a female has a bachelor’s degree, yes = 0, no = 1.

Moreover, the coefficient of "Policy" in column (4) is significantly negative and the coefficient of "Policy" in column (5) is not significant. This implies that women with a bachelor’s degree or above are not negatively affected by the UTC policy, only women without a bachelor’s degree experience a reduction in their employment income due to the UTC policy. Also, from the results in column (6), we can see that the coefficient of "Bachelor*Policy" is -0.3759 and significant at the 5% level, which means that compared to women with bachelor’s degrees, the UTC policy resulted in a 37.59% decrease in the employment income of women without bachelor’s degrees. This finding supports the aforementioned hypothesis that higher-educated women are more resilient to the negative consequences of the UTC policy. Their advanced education likely provides them with better job opportunities, higher earning potential, and greater adaptability in the labor market, which may help buffer them from the policy’s impact. In contrast, women with lower educational attainment may be more vulnerable to the UTC policy’s effects due to their limited access to higher-paying jobs and reduced labor market flexibility. The UTC policy’s implementation may lead to adjustments in their work arrangements or reduced job opportunities, resulting in a decline in their employment income.

## 6. Conclusions

Based on the panel data from five waves of the CFPS spanning from 2012 to 2020, We apply the DID model to estimate the impact of the UTC policy on women’s employment income. Additionally, we conduct several robustness tests, including Heckman Two-Step Estimation and PSM-DID, to ensure the robustness of our baseline regression results. Furthermore, we examine the mediation effect of working hours and hourly wages in the impact of the UTC policy on women’s employment income. Finally, we perform heterogeneity analysis on the baseline regression results, exploring whether the UTC policy’s effects vary across different age groups and educational backgrounds groups.

The findings of our study reveal that the implementation of the UTC policy leads to an average decrease of 20.86% in women’s employment income. We confirm the absence of selection bias and omitted variable bias in the baseline regression by using the Heckman Two-Step Estimation and PSM-DID, further validating this result. Moreover, our investigation indicates that the UTC policy achieves its negative impact by reducing women’s working hours and hourly wages, resulting in a decrease in their employment income. The UTC policy leads to an average reduction of 6.02% in women’s working hours and an average decline of 17.92% in their hourly wages. Furthermore, we find that the negative effects of the UTC policy on women’s employment income are greater among women below 35 years old and those without a bachelor’s degree.

## 7. Discussion

Our study theoretically links theories such as human capital theory, compensating wage differentials theory, and role conflict theory with the motherhood wage penalty, establishes a theoretical framework of the causes that lead to the motherhood wage penalty, and innovatively applies this theoretical framework to the analysis of the impact of the UTC policy on women’s employment income, expanding the application scenarios of these theories, which is also the theoretical implications of our study. Based on this theoretical framework, we enrich and expand the empirical research on the effects of the UTC policy. We also consider the harm to women’s interests caused by large-scale public policies by the econometric models and fill the shortcomings in the existing literature concerning gender discrimination in the labor market and female work rights. We demonstrate remarkable innovation by delving into the mediation effect of working hours and hourly wages in the impact of the UTC policy on women’s employment income. We also conduct a thorough analysis of the heterogeneity of this impact across different age groups and educational backgrounds groups of women. These analyses help to further illustrate how the UTC policy affects women’s employment income and can reflect the different responses of different types of women to the UTC policy. These findings can reflect the practical implications of our study and provide a reference for future policy reforms.

We focus on examining the impact of the UTC policy on the Chinese female labor market, revealing the dual effects of the UTC policy: while it contributes to demographic dividends, it also poses adverse consequences on the female workforce. The UTC policy appears to reinforce employers’ preference for male employees, exacerbating gender discrimination issues in the labor market, to some extent, undermining the interests of women, particularly among young women and those with lower educational attainment. The government should actively endeavor to avoid the negative impact of the UTC policy on women’s employment income, so as to foster higher fertility rates and gain better policy outcomes. This can be achieved through measures aimed at safeguarding women’s equal labor rights, bolstering labor market supervision, and combatting gender-based discrimination. Additionally, the Chinese government should encourage active male participation in childcare and household chores, so as to help alleviate the burdens on women.

While we offer a novel and insightful evaluation of the UTC policy from a unique perspective, providing fresh evidence on gender equality and female employment issues, it is also important to acknowledge some limitations. The most important limitation in our mediation analysis is that we may not have considered all the mediators. In addition to working hours and hourly wages, there may be other mediators that affect employment income, such as the attitude of the boss towards female employees, and the attitude of the husbands. However, limited by the data we have, we are currently unable to investigate the effect of these mediators, even though they are also important. Besides, the period under consideration in this study is limited to data from five waves between 2012 and 2020, possibly overlooking the long-term effects of the UTC policy. Hence, we suggest that further studies could explore the rationale behind employer preferences and provide a deeper understanding of gender discrimination in the labor market. We also believe that expanding the temporal scope in future research would offer more comprehensive and long-term insights.
